# Distribution and location of Daxx in cervical epithelial cells with high risk human papillomavirus positive

**DOI:** 10.1186/1746-1596-9-1

**Published:** 2014-01-08

**Authors:** Shuang-yang Tang, Le Li, Yao-lin Li, An-yuan Liu, Min-jun Yu, Yan-ping Wan

**Affiliations:** 1 Institute of Pathogenic Biology, University of South China, Hengyang 421001, China; 2 School of Public Health, University of South China, Hengyang 421001, China

**Keywords:** Human papillomavirus, Daxx, Cervical cancer, Intraepithelial neoplasia grade

## Abstract

**Aims:**

To provide the basis for further exploring the effect and its mechanism of Death domain associated protein (Daxx) on the progress of cervical carcinoma induced by human papillomavirus (HPV), the distribution and location of Daxx in cervical carcinoma with high risk HPV(HR-HPV) positive was analyzed.

**Methods:**

The samples of normal cervical epithelial cells, cervical intraepithelial neoplasia grade I (CINI), CINII CINIII and cervical cancers were collected. Immunohistochemistry assay was used to analyze the distributions and locations of Daxx in the cervical tissue. Indirect immunoinfluorescence test was utilized to observe the locations of Daxx in Caski cells with HPV16 positive.

**Results:**

Under the light microscopy, the brown signals of Daxx distributed in the nuclei of normal cervical epithelial cells; Daxx mainly distributed in nuclear membrane and there were a small amount of Daxx in the nuclei in CINI. Daxx intensively distributed in the cytoplasm and cell membrane in CINII, CINIII and cervical cancer. Under fluorescent microscopy, the distribution and location of Daxx in Caski cells was similarly to that in cervical cells of CINII, CINIII and cervical cancer.

**Conclusion:**

In the progress of the cervical cancer, Daxx gradually translocates from nucleus into nuclear membrane, cytoplasm and cell membrane. Daxx locates in the cytoplasm and cell membrane in CINII, CINIII and cervical cancer.

**Virtual slides:**

The virtual slide(s) for this article can be found here:

http://www.diagnosticpathology.diagnomx.eu/vs/4671548951113870.

## Introduction

Death domain associated protein (Daxx) is a highly conserved nuclear protein playing an important roles in the transcriptional control, carcinogenesis, resistance to virus infection and so on [1]. Daxx can shuttle between the nucleus and cytoplasm or other cellular substructures, suggesting it have different functions at the different cellular compartments and stages of the cell cycle. The most obvious depositories of Daxx are promyleocytic leukaemia protein (PML) nuclear bodies (PML-NBs), that is PML oncogenic domains (PODs) or nuclear domains 10(ND10) [2]. Because of many proteins locating in PML-NBs, it implies the functions of PML-NBs involve in the transcriptional regulation, growth suppression, apoptosis, and etc. The ND10-associated proteins are an important interface of viral life cycle and host cell [3]. Daxx can participate in numerous cellular functions as a mediator of protein interactions acting as a fine tuning instrument in highly orchestrated cellular processes. The accumulation of Daxx in PML NBs is as an “out of action” storage depot, however. Under the cellular stress, Daxx can interact with many kinds of molecules and affect its downstream signaling pathway [4], such as the p53/DAXX-mediated RASSF1A methylation regulating murine double minute 2 (MDM2) protein stability [5].

The incidence of cervical cancer was just less than breast cancer among all female malignant tumors in the world. More than 99.7% HPV genes can be found in cervical cancer tissues, so HPV infection was the most important factor in inducing cervical cancer, and was also the key risk factor for cervical cancer. It was found that about 70% cervical cancers were caused by HPV16 and HPV18, which both are HR-HPV [6]. About 10% HPV16 infections would be latent, despite these mechanisms are unclear, in which Daxx within PML-NBs is likely to play a role that is unclear. HPV capsid protein L2 can induce the restructuring of PML-NBs, and increase the gathering of Daxx co-locating with PML [7]. HPV18 E6 can co-locate with PML and promote its degradation by proteasome pathway, and inhibit the cell aging caused by PML [8]. In addition, HPV E1, E2, E5, E6, E7 and L1 are associated with PML-NBs being the originating parts of the HPV infections [2]. Therefore, Daxx locating in PML-NBs may play a very important role in intracellular defense against viral infection.

In our previous report, we found that HPV16 E6 could translocate some Daxx from the nucleus to the cytoplasm when HPV16 E6 over-expressed in HeLa cells [9], which might be related to the normal form change of PML-NBs. If Daxx were transferred from nucleus to the cytoplasm, its functions should be affected. After HPVs infect cervical epithelial cells, Daxx may interact with E6, E2 or other viral proteins, then directly affect the HPV DNA replication and E6/E7 transcription. It is supposed that in cervical tissue cells infected by HPVs, early or late viral protein may influence the distribution of Daxx, its subcellular localization, and its post-translational modification, accordingly the function of Daxx may be changed. Therefore, in this report we study the locations of Daxx in the different stages of cervical epithelial cells with HR-HPV positive from normal cervical tissue to cervical cancer and in Caski cells with HPV16 positive, so as to confirm the expression and localization of Daxx are changed in these cervical epithelial cells.

## Materials and methods

### Patients and tissue samples

The procedure for this research project conforms to the provisions of the Declaration of Helsinki. All the cervical tissue specimens being obtained from patients with HR-HPV were collected from Hengyang Women and Children Health Hospital, Hengyang, Hunan, China and 169^th^ Hospital of Chinese People’s Liberation Army, Hengyang Hunan, China. These specimens had been stored as paraffin-embedded sections. All specimens had been analyzed for Daxx protein expression using the immunohistochemistry assay.

### Cells and reagents

Caski cells with HPV16 positive were provided by Institute of pathogenic biology, university of South China. Rabbit anti-human Daxx antibody was purchased from Santa Cruz (Santa Cruz, CA, USA). HRP-Goat Anti-Rabbit IgG (HRP-GAR) and FITC-Goat Anti-Rabbit IgG (FITC-GAR) were bought from Sigma (Santa Clara, CA, USA). Avidin–Biotin Complex (ABC) Vectastain Kit was obtained from Vector Laboratories (Burlingame, CA, USA).

### Immunohistochemistry assay

Immunohistochemistry assay was carried out using the Avidin–Biotin Complex (ABC) Vectastain Kit according to the manufacturer’s instructions. At first, the cervical cancer tissue pathological slices were soaked with xylene for 10 min, then xylene was abandoned and the above process was repeated once. Second, all slices were soaked with absolute ethyl alcohol for 5 min, following with 95% ethyl alcohol and 70% ethyl alcohol repeating above process, then were washed by PBS three times for 5 min at every time. Furthermore, the slices were heated in Sodium citrate buffer solution (pH6.0) at 95°C for 10~15 min and washed normally. Then the sections were blocked with blocking buffer containing 5% BSA at 37°C for 20 mins. Sections were then incubated with rabbit anti-human Daxx antibody and then with secondary antibody. Finally, the sections were incubated with avidin–biotin complex reagent before being stained with diaminobenzidine and counterstained with Mayer’s hematoxylin. All of the stained sections were evaluated in a blinded manner without prior knowledge of the patient data.

### Indirect immunofluorescence assay

Caski cells were cultured in RPMI-1640 medium contained 10% fetal bovine serum (FCS) at 37°C. When the celluar density reached about 50%, the cells growth solution was discarded, and then the cells were washed twice with PBS, which were done after the later every one treatment. After the adding of 4% triformol to fix for 10 mins, 0.2% of Triton X-100 was added to react for 10 mins. Block was processed with 2% of bovine serum albumin for 1 h, following by the incubation overnight with Daxx antibody. Then the mixture of FITC-GAR and DAPI was added at 37°C for 1 h. After the last washing, the supernatant was discarded. Finally, the cells were observed under inverted fluorescence microscope.

## Results

### Expression of Daxx in cervical cells infected by HR-HPV

**Figure 1:**
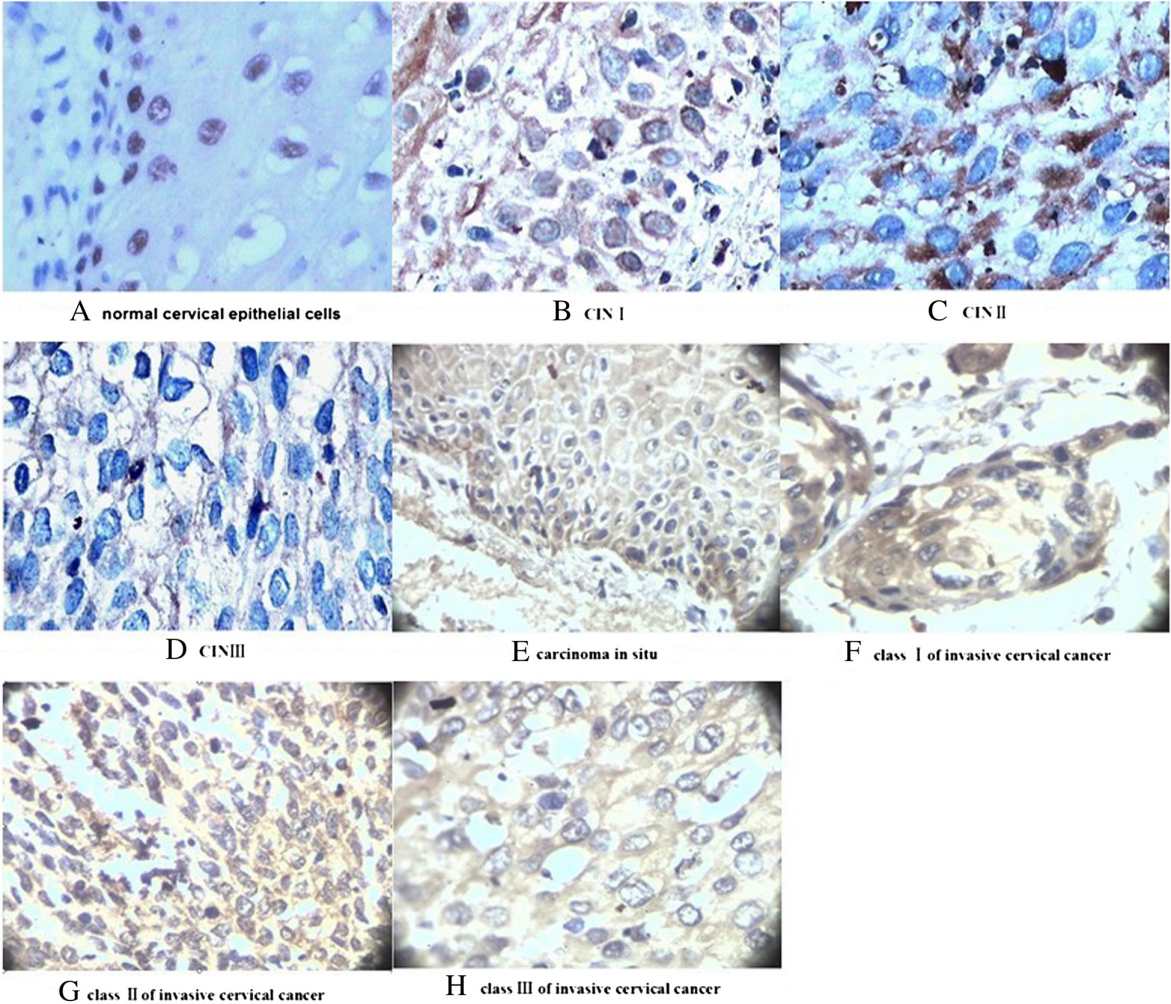
**Distribution and location of Daxx in cervical epithelial cells (10 × 40). A:** All Daxx existed in nucleus in the normal cervical epithelial cells; **B:** Daxx mainly distributed nearby the nuclear membrane, a little within the nucleus in HR-HPV positive cells of CIN I; **C, D, E, F, G, H:** Daxx densely distributed in the cytoplasm and cell membrane in HR-HPV positive cells of CINII, CINIII, carcinoma in situ and invasive cervical cancer.

Under the optical microscope, Daxx was tan in cervical tissue using immunohistochemical test (Figure 1). In normal cervical epithelial cells, Daxx distributed in the nucleus, and only located in the nucleus (Figure 1A); in HR-HPV positive cells of CIN, Daxx mainly distributed nearby the nuclear membrane, a little within the nucleus (Figure 1B); in HR-HPV positive cells of CIN, CIN, carcinoma in situ and invasive cervical cancer, Daxx densely distributed in the cytoplasm and cell membrane (Figure 1C, D, E, F, G, H).

### Location of Daxx in Caski cells

**Figure 2:**
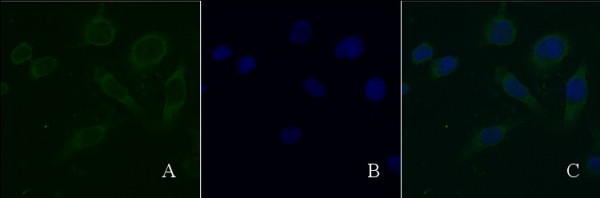
**Localization of of Daxx in Caski cells(10 × 100). (A)** Caski cells were immunostained with Rabbit anti-Daxx antibodies visualized with a Cy2-conjugated goat anti-rabbit IgG (green). **(B)** Caski cells were immunostained with DNA dye (blue). **(C) (A)** and **(B)** were overlapped.

Under the fluorescent microscopy, the blue fluorescence was assigned to the nucleus. The fluorescence of Daxx (green) was mainly distributed nearby the nuclear membrane of Caski cells with HPV16 positive, of which a little was distributed in the nucleus (Figure 2). The distribution and location of Daxx in Caski cells with HPV16 positive was similarly to that in cervical cells of CINII, CINIII and cervical cancer.

## Discussion

Daxx is one of the few “constitutive” components of PML-NBs, and is a multifunctional protein that regulates a wide range of cellular signaling pathways for both cell survival and apoptosis. It is a critical regulator of T-lymphocyte homeostasis by decreasing TCR-induced cell proliferation and by promoting Fas-mediated cell death [10]. Daxx has been shown that it acts as a scaffold that stimulates the phosphorylation of p53 by the protein kinase Hipk2 [11]. As a novel APC/C inhibitor frequently overexpressed in prostate cancer, Daxx was frequently upregulated in prostate cancer tissues, and its expression level positively correlated with the Gleason score and disease metastasis. Furthermore, its ectopic expression in a non-malignant prostate epithelial cell line induced polyploidy under mitotic stress. Daxx may promote chromosome instability during prostate cancer development [12]. So it implies Daxx might play a critical role in the cellular transformation and cancer development.

Daxx’s location within PML-NBs is crucial for normal cellular shapes and functions. Under some influencing factors, the subcellular localization of Daxx can be changed by modification or interacting with other proteins. The changing of Daxx location can affect the cell cycle, the antiviral, pro-apoptotic or antiapoptotic activities and play a role in the transcriptional regulation [2]. In recently study on brain tissue infected by reoviridae, it was found that the up-regulation of Daxx through IFN-I mechanism might depend on the Daxx positioning cytoplasm or nucleus to play a role in cell apoptosis [13] which showed that Daxx orientation was related to the apoptosis role of host cell after viral infections. In Human cytomegalovirus (HCMV)-infected cells, Daxx was bound to HCMV virion tegument protein pp71, which also promoted its sumoylation [14]. If HCMV didn’t express the protein IE1, coat protein pp71 in cytoplasm didn’t combine with Daxx, therefore Daxx would silence the viral genes and inhibit the viral replication. But the newly synthesized pp71 protein could enter into the nucleus to interact with Daxx and reverse inhibition mediated by Daxx, which was advantageous to the viral genes expression [15]. But later it was found that HCMV infected cells could close the mRNA expression of viral early protein gene IE1 and IE2 at S/G2 phase, which didn’t rely on the functions of Daxx and PML. So, the nuclear, genome-wide repression of HCMV was typically mediated by the intrinsic immune defence at ND10 structures [16]. Adenovirus(Ad) E1B-55kd protein can also interact with Daxx and break the colocalization of Daxx and PML within PML-NBs [17], so affect the transcription inhibitory activities of Daxx and its other functions [18]. It was shown that the protein expression of chromatin remodeler Daxx in high grade invasive urothelial carcinoma of the bladder and in its preinvasive phases changed through quantitative immunohistochemical analysis [19]. Which effects have Daxx in the progress of cervical cancer induced by HR-HPVs?

Like adenovirus, HPVs replicate in the nucleus. We speculate that after HPVs infections in cervical epithelial tissue, the normal shape of PML (NBs) might be changed and following the location changing of Daxx, furthermore it would affect the cellular resistance for the HPVs. Owing to the HPVs persisted infections in cervical epithelial tissues, cervical cancer would happen eventually. In this study we found all Daxx existed in nuclei of the normal cervical epithelial cells. however, in HR-HPV positive cells of CIN, majority of Daxx already transfered from the nuclei and gathered near the nuclear membrane, but a little existed in nuclei, which was further confirmed by the consistance of its loation in Caski cells with HPV16 positive. With the hyperplasia and deterioration of epithelial tissue, Daxx translocated through nuclear membrane into the cytoplasm, and intensive distribution in the cell membrane. This study also confirmed that the translocated process is completed in CIN, due to Daxx distribution and localization in CIN were all identity with that in CIN, carcinoma in situ and invading cervical cancer tissues. Our study results suggested that Daxx might play an important role in the antineoplastic early stage. While the host cells had been immortalized, Daxx would play a little role in antitumor.

Owing to the obvious location changing of Daxx between in CINI and CINII, it may be an important target protein for protecting cervical tissue against HPVs infections inducing cervical cancer. In the cervical epithelial cells persistently infected by HPVs, if Daxx translocating from the nucleus to the cytoplasm can be intervened, it would be possible to avoid cells immortalization, and prevent the cervical cancer induced by HR-HPVs.

## Competing interests

The authors declare that they have no competing interests.

## Authors’ contributions

ST and LL constructed the manuscript. ST and YL carried out immunohistochemical assay. AL and MY carried out pathologic study, LL and ST carried out indirect immunofluorescence assay, YW designed the study and reviewed the manuscript. All authors read and approval the final manuscript.

## References

[B1] Yang X Khosravi-Far R Chang HY Baltimore D Daxx, a novel Fas-binding protein that activates JNK and apoptosis Cell 1997 89 1067 1076 10.1016/S0092-8674(00)80294-9 9215629 PMC2989411

[B2] Lindsay CR Morozov VM Ishov AM PML NBs (ND10) and Daxx: from nuclear structure to protein function Front Biosci 2008 13 7132 7142 18508722 10.2741/3216

[B3] Florin L Schäfer F Sotlar K Streeck RE Sapp M Reorganization of nuclear domain 10 induced by papillomavirus capsid protein l2 Virology 2002 295 1 97 107 10.1006/viro.2002.1360 12033769

[B4] Chen SF Zhu CM Wan YP Progress on the subcellular localization of Daxx Ai Zheng 2009 28 1333 1336 19958631 10.5732/cjc.009.10168

[B5] Zhang H He J Li J Tian D Gu L Zhou M Methylation of RASSF1A gene promoter is regulated by p53 and DAXX FASEB J 2013 27 1 232 242 10.1096/fj.12-215491 23038753 PMC3528318

[B6] Schiller JT Lowy DR Prospects for cervical cancer prevention by human papillomavirus vaccination Cancer Res 2006 66 21 10229 10232 10.1158/0008-5472.CAN-06-0630 17079437

[B7] Lin Z Yemelyanova AV Gambhira R Jagu S Meyers C Kirnbauer R Ronnett BM Gravitt PE Roden RBS Expression pattern and subcellular localization of human papillomavirus minor capsid protein L2 Am J Pathol 2009 174 1 136 143 10.2353/ajpath.2009.080588 19095951 PMC2631326

[B8] Guccione E Lethbridge KJ Killick N Leppard KN Banks L HPV E6 proteins interact with specific PML isoforms and allow distinctions to be made between different POD structures Oncogene 2004 23 27 4662 4672 10.1038/sj.onc.1207631 15107834

[B9] Chen SF Zhu CM Tang SY Yu MJ Su SM Yang F Wan YP Localization of human papillomvirus type 16 E6 protein and hDaxx in a human cervical carcinom cell line HeLa and their effects on cell apoptosis Chinese J Dermatol 2012 45 400 403

[B10] Leal-Sanchez J Couzinet A Rossin1 A Abdel-Sater F Chakrabandhu K Luci C Anjuere F Stebe E Hancock D Hueber AO Requirement for Daxx in mature T-cell proliferation and activation Cell Death Differ 2007 14 4 795 806 10.1038/sj.cdd.4402056 17082815

[B11] Li Q Wang X Wu X Rui Y Liu W Wang J Daxx cooperates with the Axin/Hipk2/p53 complex to induce cell death Cancer Res 2007 67 66 74 10.1158/0008-5472.CAN-06-1671 17210684

[B12] Kwan PS Lau CC Chiu YT Man C Liu J Tang KD Wong YC Ling MT Daxx regulates mitotic progression and prostate cancer predisposition Carcinogenesis 2013 34 4 750 759 10.1093/carcin/bgs391 23239745

[B13] Dionne KR Zhuang Y Leser JS Tyler KL Clarke P Daxx upregulation within the cytoplasm of reovirus-infected cells is mediated by interferon and contributes to apoptosis J Virol 2013 87 6 3447 3460 10.1128/JVI.02324-12 23302889 PMC3592169

[B14] Hwang J Kalejta RF In vivo analysis of protein sumoylation induced by a viral protein: detection of HCMV pp 71-induced Daxx sumoylation Methods 2011 55 2 160 165 10.1016/j.ymeth.2011.07.004 21816224 PMC3208771

[B15] Hwang J Kalejta RF Proteasome-dependent, ubiquitin-independent degradation of Daxx by the viral pp 71 protein in human cytomegalovirus-infected cells Virology 2007 367 334 338 10.1016/j.virol.2007.05.037 17590404

[B16] Zydek M Uecker R Tavalai N Stamminger T Hagemeier C Wiebusch L General blockade of human cytomegalovirus immediate-early mRNA expression in the S/G2 phase by a nuclear, Daxx- and PML-independent mechanism J Gen Virol 2011 92 2757 2769 10.1099/vir.0.034173-0 21832009

[B17] Zhao LY Colosimo AL Liu Y Wan Y Liao D Adenovirus E1B 55-kilodalton oncoprotein binds to Daxx and eliminates enhancement of p53-dependent transcription by 18 Daxx J Virol 2003 77 11809 11821 10.1128/JVI.77.21.11809-11821.2003 14557665 PMC229361

[B18] Schreiner S Wimmer H Sirma H Proteasome-dependent degradation of Daxx by the viral E1B-55 K protein in human adenovirus-infected cells J Virol 2010 84 7029 7038 10.1128/JVI.00074-10 20484509 PMC2898266

[B19] Zizzi A Montironi MA Mazzucchelli R Scarpelli M Lopez-Beltran A Cheng L Paone N Castellini P Montironi R Immunohistochemical analysis of chromatin remodeler Daxx in high grade urothelial carcinoma Diagn Pathol 2013 8 111 10.1186/1746-1596-8-111 23819605 PMC3751668

